# The Wither or Thrive Model of Resilience: an Integrative Framework of Dynamic Vulnerability and Resilience in the Face of Repeated Stressors During the COVID-19 Pandemic

**DOI:** 10.1007/s42844-022-00069-7

**Published:** 2022-07-13

**Authors:** Malvika Godara, Sarita Silveira, Hannah Matthäus, Tania Singer

**Affiliations:** Social Neuroscience Lab, Max Planck Society, JFK Haus, Bertha-Benz-Str. 3, 10557 Berlin, Germany

**Keywords:** Psychological resilience, Vulnerability, COVID-19, Risk, Processing mechanisms, Social cohesion, Mental health

## Abstract

During the first 2 years of the COVID-19 pandemic, empirical efforts in the psychological sciences have been unequivocally focused on understanding the psychosocial impact on resilience and vulnerability. While current empirical work is guided by different existing theoretical models of resilience and vulnerability, the emerging datasets have also pointed to a necessity for an update of these models. Due to the unique features and developments specific to the current pandemic such as the occurrence of repeated collective stressors of varying durations, in the current position paper, we introduce the Wither or Thrive model of Resilience (With:Resilience). It integrates key aspects of prevailing psychological resilience frameworks within the context of the COVID-19 pandemic, and extends them by (1) moving away from single scale approaches towards a higher-order latent expression of resilience and vulnerability incorporating also non-clinical mental health markers, (2) proposing different trajectories of resilience-vulnerability emerging across repeated stressors over long periods of time, and (3) by incorporating multiple influencing factors including aspects of the socio-economic concept of social cohesion as well as separate mediating processing mechanisms. We propose that With:Resilience will enable a more nuanced approach and appropriate analytical investigation of the vast incoming data on mental health and resilience during the COVID-19 pandemic, and we suggest some concrete methodological approaches. This framework will assist in the development of actionable public health guidelines for society in the present and future pandemic contexts as well as aid policy making and the interventional sciences aimed at protecting the most vulnerable amongst us.

## Introduction

The SARS-CoV-2 pandemic has had an immense impact on the daily lives of people across the globe. A vast empirical effort has been launched by researchers across the globe to document and understand the psychological impact, in terms of mental health and psychologically resilient functioning, resulting from the pandemic. While there exists an abundance of theoretical and empirical models of resilience and vulnerability in the field of psychology (Block & Block, 1980; Kalisch et al., 2015; Masten et al., 2021a, 2021b), COVID-19 pandemic-specific developments, such as exposure to repeated collective stressors for entire populations simultaneously, necessitate an update of previous models. A multitude of pandemic-related mental health studies are emerging that cannot be easily understood, statistically modelled and accounted for by previous resilience models, which remain still rather fragmented in terms of orientations and are unable to fully account for the effects of such repeated, collective and long-lasting stressors such as multiple lockdowns. The Wither or Thrive model of Resilience or the With:Resilience model seeks to provide such an integrative framework by bringing together and extending several different existing perspectives on stressor-related psychological resilience and vulnerability that are existing in the field with the goal to be better applicable to the current pandemic context and allowing for a comprehensive understanding and modelling of the empirical findings pertaining to resilience and vulnerability during the continued COVID-19 pandemic. The framework extends the previous models within the pandemic context in several ways. First, within the With:Resilience framework, psychological resilience and vulnerability are posited to be two dimensions of a higher-level latent construct based on multiple indicators including stress, loneliness, and other vulnerability markers not exclusively reserved to clinical outcomes such as depression. Second, it is proposed that these outcomes can be modeled as dynamic time courses extending over a longer time period extending over several years and including the presence or absence of multiple collective stressors such as lockdowns of different duration, leading to emergence of unique trajectories of resilience-vulnerability. Third, it further suggests a combination of different categories of influencing factors ranging from individual biological and psychological factors such as genetic markers like FKBP5 or psychological factors like neuroticism, individual contextual- and demographic factors such as gender or household composition, to intersubjective, social factors such as empathy or sense of belonging, all of which act as protective or risk factors. Fourth, it also proposes a category of processing mechanisms such as epigenetic changes like DNA methylation or cognitive-behavioral coping and emotion regulation strategies such as acceptance, which exert the influence of predictors on resilience-vulnerability. Finally, the framework endeavors to bring together the various domains in which psychological resilience and vulnerability can be manifested during the present pandemic context, including neuroscience, biology, psychology, and social sciences. Since the With:Resilience model is highly dynamic in nature and endeavors to take a comprehensive and an all-encompassing approach to time-varying and context-dependent influencing factors as well as person-specific characteristics assessed at a given point in time and development, the framework could be applied across the developmental spectrum and could be useful for studying resilience-vulnerability processes in children and adolescents just as in adults.

In the present paper, we will begin by providing the rationale for a pandemic-specific framework for resilience-vulnerability in relation to the psychological impact of the COVID-19 pandemic in the first section. This will be followed by a section giving a brief overview of the current state of the art in the field of resilience, describing the various prevailing models of resilience-vulnerability. This section will continue to explain how the With:Resilience framework integrates the various features of the current models of resilience-vulnerability, followed by an overview of the limitations of these models in the present pandemic context and how the pandemic-specific With:Resilience framework seeks to fill these gaps. The next section of the paper will explain the main features of the With:Resilience, followed by subsections detailing the various predictor and mechanism categories. This will be followed by a final section describing various perspectives surrounding the With:Resilience model, namely the methodological recommendations, empirical considerations, and the practical and policy implications emerging from the framework.

## The Psychological Impact of the Pandemic

In addition to the stress and bereavement caused by contracting the disease or losing a close one to it, the COVID-19 pandemic created and amplified socioeconomic adversities due to job loss or financial insecurity as well as psychological burden through persistent negative news and multimedia access to negative content. In order to control the spread of the COVID-19 disease, governments across the world have prescribed public health measures such as lockdowns and stay-at-home orders which required people to socially isolate. Such unprecedented social isolation and disruption of the daily functioning of people has created further multiple psychological challenges. Consequently, a growing body of research has shown debilitating effects on psychological functioning in the general public during the pandemic, with existing cohort studies highlighting the increase in difficulties from pre-pandemic levels (Tsamakis et al., 2021; Vindegaard & Benros, 2020). Countless empirical studies have depicted increasing levels of depression, anxiety, subjective stress, and mental burdens in the general public during the course of the pandemic (Fancourt et al., 2021; Li et al., 2020; Moccia et al., 2020), and also in comparison to pre-pandemic levels (Kwong et al., 2021; Pierce et al., 2020). Several studies have pointed out the increased vulnerability to mental health problems in groups such as younger individuals (18–29 years age), women and unemployed individuals (Gloster et al., 2020; Huang & Zhao, 2021; Loades et al., 2020). Furthermore, specific contextual factors such as being a system-relevant or essential worker or being a caregiver showed further poorer mental health outcomes such as increased psychological distress and poor sleep quality (Cai et al., 2020; Racine et al., 2021; Xu et al., 2020). Moreover, contracting the COVID-19 disease also led to increased reporting of depressive and post-traumatic stress symptoms. There is also accumulating evidence suggesting the increased use of maladaptive coping styles, such as increased alcohol abuse and aggressive tendencies in the form of domestic violence (Gautam et al., 2020; Moreno et al., 2020). Therefore, a plethora of research has emerged investigating poor mental health as a result of the COVID-19 pandemic, and identifying the individuals who are at a greater risk of developing mental disorders as an outcome of the repeated collective stressor of the pandemic.

A parallel line of empirical work has been focused on understanding how levels of psychological resilience and people’s ability to cope has been affected due to the pandemic. Studies have reported lower levels of resilience during the pandemic, specifically these low levels of resilience have been found in individuals who also reported more negative mood and other mental health difficulties during the pandemic (Blanc et al., 2021; Killgore et al., 2020). Several studies have also investigated the use of a variety of coping and emotion regulation strategies to cope with the distress due to the pandemic. Accordingly, studies have shown that in the initial months of the pandemic, people engaged in regulation and coping strategies such as rumination (Petzold et al., 2020) and worry (Killgore et al., 2020) by consuming negative media content related to the pandemic, and they were negatively linked to psychological resilience and led to increased mental health issues. However, increasingly studies also started pointing out the various coping strategies that could serve as potential protective factors or mechanisms that promote psychological resilience. As such, studies found that individual and social resources such as psychological flexibility, social support, acceptance, use of religion, and spending time in nature and physical exercise could all buffer adverse effects on mental health and increase resilience (Chong et al., 2021; Gloster et al., 2020; Pakenham et al., 2020). Therefore, this parallel line of research has outlined that risk and protective factors can lead to differential patterns of psychological vulnerability and resilience during the pandemic, with some factors leading an individual to having more mental health difficulties and disposing them to the development of psychological disorders during the pandemic while other factors leading another to be resilient.

The number of empirical studies reporting poor mental health and resilience during the pandemic are accruing with speed and they are certainly the necessary first step in the process of documenting the psychological impact of the pandemic. However, to be able to comprehensively process the extent and magnitude of the mental health crisis brought on by the pandemic and the related individual shift towards vulnerability or resilience, the entire body of these empirical studies needs to be converged in a unified manner. However, the current empirical work emerging on mental health and resilience during the pandemic remains rather scattered due to the broad range of scope and methodology being applied across the studies. As such, there arises a need for a theoretically-driven framework that can facilitate an integrated and comprehensive understanding of the scale and magnitude of the pandemic-related impact on mental health and psychological resilience. Moreover, as the nature of the pandemic itself is evolving, and along with it the public health responses, the empirical methods and study procedures will also continue to adapt and evolve. In view of this, such a theoretically driven framework will also be imperative to guiding a directed and focused empirical endeavor. Furthermore, one of the important objectives of the research aimed at understanding the psychological impact of the pandemic is to be able to identify the specific factors and areas which could benefit from interventions to support better mental health. For example, when an empirical study shows that women or young people are especially psychologically vulnerable during the pandemic, it becomes clear that special interventions need to be applied to better support the mental health of these groups. However, given the wide range of protective and risk factors emerging for mental health and resilience during the pandemic, including socio-demographic, contextual or individual factors, it becomes unclear how and when intervention support needs to be provided to various vulnerable groups. A theoretically-driven integrative framework will be able to provide the filtering process through which it can be empirically clarified which individuals and groups need to be especially supported at which time points before, during and after situations of collective and repeated stressors such as the pandemic and related lockdowns. Lastly, a theoretically driven framework will be able to provide a foundation for the generation of empirically-derived policy implications. While various empirical studies discuss and recommend a variety of ramifications for policies and policy-makers, these implications often remain broad in purview and vague in application. A holistic framework that helps organize the empirical base will be able to provide space for targeted and precise guidelines for policymakers and organizations to best support the populace during repeated and collective stressors. Therefore, in this paper, we introduce the With:Resilience framework, in an endeavor to provide such a theoretically-guided comprehensive framework. While there is an existence of a range of theoretical models of resilience that provide an understanding of how psychological resilience is manifested in everyday life and in stress situations, the With:Resilience framework integrates these perspectives in a unified manner. Despite that, we observe that within the current pandemic context, the prevailing models of resilience have recognizable limitations to explaining resilience-vulnerability processes to the most nuanced and broad extent. Therefore, in the next section, we provide a brief overview of the key models of resilience-vulnerability prevalent in the field, and how our proposed framework will convene these viewpoints into a more integrated perspective. Importantly, we will discuss the limitations of these models in the current pandemic context and how the With:Resilience framework can address these gaps.

## State of Art in the Field of Resilience

Psychological resilience is seen as the ability of an individual to adapt to stress and adversity and is often regarded as the ability to cope and positively adapt despite experiences of significant trauma or adversity (Fletcher & Sarkar, 2013; Vella & Pai, 2019). Taking from the community resilience perspective, the capacity of disaster-affected communities to bounce back from the adverse event, natural or man-made disaster, is characterized as disaster resilience (Aldrich & Meyer, 2015; Alexander, 2013; Manyena, 2006). Accordingly, psychological resilience is also frequently defined as the ability to bounce back and recover from stressful experiences (Bonanno, 2004; Bonanno & Diminich, 2013; Campbell-Sills & Stein, 2007). Different schools of thought have conceptualized resilience differently, with primarily four main orientations emerging: trait resilience, resilience as an outcome, resilience as a process, and a multisystem resilience perspective. Trait resilience views resilience as an innate and personal characteristic of an individual to successfully cope with stressors and manage adjustment and functioning in the aftermath of stressor exposure (Fletcher & Sarkar, 2013; Hu et al., 2015). From this view, resilience, much like personality, is considered to be an individual trait comprised of various components, the presence of which leads an individual to be resilient to any adversity or stressor (Connor & Davidson, 2003; Ong et al., 2006; Waugh et al., 2011). Researchers conceptualizing trait resilience have identified several different constructs, such as competence and resourcefulness, that comprise this innate ability to adapt and thrive in the face of adversity (Waugh et al., 2011). On the other hand, researchers viewing resilience as an outcome, characterize resilience as a product of interaction between various individual attributes and stressful events (Harvey & Delfabbro, 2004). Here, the positive outcomes during the post-stressor period, and the recovery to pre-stressor functioning are considered to be resilient responses (Masten, 2001; Vella & Pai, 2019). Relatedly, the stress-inoculation perspective explains resilience as a by-product of previous adversities and individual factors that have led to the building of individual capacities geared towards resilient adaptation and recovery in the face of future stressors (Seery & Quinton, 2016). These perspectives indicate that resilience could potentially be stemming from an interaction between individual, contextual and environmental factors.

Along these lines, resilience is now predominantly being viewed as a dynamic process of coping that is influenced by multiple independent individual and environmental factors (Bonanno et al., 2011). This has led to the identification of several different trajectories of resilient functioning and maladaptive development following a stressor (Bonanno, 2004). A key contribution of this line of research has been the distinction between a trajectory of resilience and a trajectory of recovery, along with the idea that adaptive and maladaptive functioning as a function of stressor exists on a continuum (Bonanno, 2004; Galatzer-Levy et al., 2018). This has further led to the emergence of the idea that resilience exists as a dynamic process of adaptation that is reflected in how well an individual is able to cope with a stressor and the levels of positive mental health outcomes (Kalisch et al., 2017; Stainton et al., 2019). From this perspective, individual, contextual, and environmental factors themselves are not indicators of presence or absence of resilience, but in fact they are potential protective or risk factors that influence the dynamic process of adaptation to the stressor, and thereby a trajectory of resilience. Moreover, this approach classifies appraisal of the adverse or stressful situation in its entirety, comprising as a resilience mechanism through which the resilience factors assert influence over the dynamic resilience process (Kalisch et al., 2015). Importantly, this perspective highlights the distinction between resilience factors and resilience mechanisms. Resilience factors could be seen as trait predispositions, such as optimism, mastery, or resourcefulness, that could make an individual more or less likely to responding to a stressor in a certain resilient or maladaptive manner. On the other hand, resilience mechanisms are the processes that allow an individual to actively adapt in the face of adversity, such as engagement of appropriate coping skills such as acceptance of difficult emotions or positively re-appraising difficult situations. Both resilience factors and mechanisms work in tandem and are highly dependent on the nature of the stressor itself, indicating the influence of the internal and external context on the dynamic resilience process. Along these lines, the multisystems approach postulates resilience to be spread out across various internal and external systems that interact with each other to yield individual resilience (Liu et al., 2017; Masten et al., 2021a, 2021b; Panter-Brick & Leckman, 2013; Ungar, 2018). Deriving from a developmental perspective, the multisystem approach conceptualizes resilience as the ability of a dynamic system of interconnected processes to adapt to stressor-related disruptions in functioning and development (Masten et al., 2021a, 2021b). These interconnected processes exist in the form of network clusters of individual, family, community, interpersonal and socio-ecological factors, each of them forming a unique complex system. These systems then interact with and influence each other to generate a dynamic resilience process that manifests when challenges and difficulties occur.

Taken together, these different approaches view resilience as a dynamic process associated with the interaction of networks of various trait-like individual, external, and mechanistic factors that potentially function as broad systems that are interconnected. The facets of the larger socio-ecological context, which includes the stressor event itself, also form a part of this dynamic process not only by the way of initiating a challenge to normal function but also through their interconnection with the other internal and external systems.

## Limitations, Integration, and Extension

**Fig. 1:**
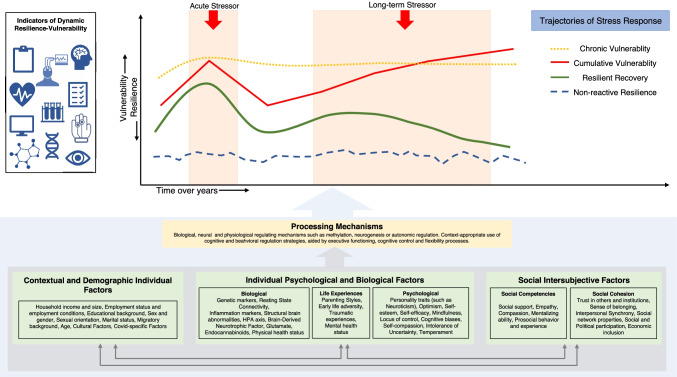
The With:Resilience model. The graph depicts the four trajectories of resilience-vulnerability that are proposed to emerge as a result of acute and chronic stressors over time. *y*-Axis indicates the levels of resilience and vulnerability and *x*-axis represents the evolution of time. Indicators of dynamic resilience-vulnerability could be biological, physiological, neuroscientific, cognitive, behavioral, self-report, or interview measures. The blue box underneath the graph consists of the three categories of predictors of resilience-vulnerability trajectory: individual psychological and biological factors, contextual and demographic individual factors, and social intersubjective factors. These predictors are considered to be interacting with each other. A category of processing mechanisms mediates the influence of these predictors on the outcome of resilience-vulnerability at any given timepoint and on the dynamic trajectory over time

The With:Resilience model seeks to harness this shared understanding of different approaches of resilience to be able to apply it to the current COVID-19 pandemic. In line with these models, the With:Resilience model also posits that psychological resilience is reflected in a dynamic process that leads to adaptation in the face of adversity (Chen & Bonanno, 2020; Masten & Motti-Stefanidi, 2020). Importantly, in the With:Resilience model, we propose a set of distinct trajectories of resilience-vulnerability that could potentially emerge in the pandemic context. Figure 1 illustrates examples for such dynamic time courses extending over longer duration of the presence and absence of collective stressors such as lockdowns of different durations. Furthermore, nearly all prevailing views surrounding resilience universally suggest a variety of independent and interacting factors that predict the course of the resilience process and the resulting trajectories. Similarly, the With:Resilience model aims at integrating different separable categories of influencing factors and suggest a range of individual biological and psychological, demographic and contextual, and social intersubjective factors that predict resultant dynamic resilience-vulnerability. Importantly, With:Resilience provides a separate set of modulatory mechanisms that further exert and mediate the influence of predicting factors on the dynamic resilience-vulnerability process over time. This focus on the factors that predict the resilience-vulnerability outcomes and the process over time, and the mechanisms through which these predictors function, also highlights how such a model can be helpful in understanding the interindividual variability in the scope of the pandemic-related impact on mental health through outlining distinct trajectories of resilience-vulnerability (Ahrens et al., 2021). We elaborate on these shared integrative aspects of the With:Resilience model, and how they are conceptualized and proposed to function in our framework, in the next section.

Extending these models, we further discuss several aspects that are specific to the COVID-19 pandemic and inhibit the holistic application of the prevailing models to the current context. Accordingly, a majority of the current models conceptualize resilience within the purview of traumatic events and post-traumatic trajectories (Bonanno, 2004; Kalisch et al., 2015), or from the developmental psychopathology perspective of early life adversity or childhood maltreatment (Garmezy, 1974; Masten et al., 2021a, 2021b). While for many of those who lost loved ones to the COVID-19 disease or for healthcare workers, the current pandemic can indeed be considered a (potentially) traumatic event, for the broader general population the pandemic can be best characterized as a prolonged period that includes the accumulation of several distinct stressor events of different duration over a long period of time. Therefore, neither an early-life adversity approach where the stressor was in the early childhood and frequently retrospectively assessed nor a trauma perspective based on past single traumatic events to resilience might be able to fully capture the data emerging from the worldwide COVID-19 pandemic now. Another stream of research which better captures the effects of present-moment collective stressors on individuals or a whole population at once and also comes with generalized socio-economic fears or circumstantial uncertainty is natural disaster research. Deriving from the community resilience perspective that has emerged in the wake of natural and man-made disasters (López-Ibor, 2006; Math et al., 2015), studies examining longitudinal impact of disasters have primarily focused on evaluating trajectories of resilience-vulnerability owing to acute-onset natural disasters (Galea et al., 2008; Kristensen et al., 2009; Mandavia & Bonanno, 2019; Pietrzak et al., 2012; Weems et al., 2010). However, acute-onset natural disasters are a conceptually distinct phenomenon from the present COVID-19 pandemic as they tend to be shorter in duration unlike the present pandemic which has morphed drastically over years since its inception in 2020. Moreover, natural disasters, such as earthquakes or hurricanes, are normally associated with a specific area and do not impact on a global scale as the current pandemic has. With respect to biological disasters and epidemics, such as the severe acute respiratory syndrome (SARS) epidemic of 2002–2004, a similar focus has been maintained on examining resilience processes during the outbreak and the trajectories of resilience-vulnerability in the aftermath (Bonanno et al., 2008).

Many lessons have been especially learned from the empirical studies on resilience-vulnerability during the SARS epidemic and the associated models of resilience that emerged then, and they have been useful in managing the initial psychological impact of the COVID-19 pandemic, specifically in Asian countries (Hsieh et al., 2021). However, this perspective does not fully encompass the resilience-vulnerability processes occurring during a prolonged pandemic over years. Despite the many similarities between the SARS epidemic and the COVID-19 pandemic, with both being respiratory infections, the infection burden and the death toll however have been significantly more pronounced and at a larger scale in the COVID-19 pandemic (Hsieh et al., 2021). Therefore, although the knowledge accrued from prior biological disasters is essential to our understanding of how mental health and resilience will be affected and expressed in the current pandemic, the implementation of these resilience models needs to be adapted to the current context (Hsieh et al., 2021). Importantly, the nature of the COVID-19 pandemic is evolving. With new variants emerging and multiple waves of infections and deaths taking place, lockdowns to curb the numbers have been imposed repeatedly. As such, the current frameworks which only account for an acute outbreak event, and its consequent aftermath, fall short of being able to capture the full extent of the dynamic resilience-vulnerability process during the COVID-19 pandemic lasting over couple years. Crucially, the nature of stressors within the current pandemic is repetitive due to the recurrent imposition and withdrawal of lockdowns and social isolation measures, mimicking almost an artificial application and removal of the same stressor multiple times. This repeated aspect of the same stressor is not sufficiently reflected in the current models of resilience, even when considering chronic stressor perspectives (Vins et al., 2015). As such, resilience frameworks that examine how dynamic resilience processes are initiated during a chronic event, and develop in the aftermath, will fall short when the same stressor is repeated and perhaps the trajectory changes course during those successive iterations. Additionally, it is also a possibility that the various lockdowns have been experienced differently for various reasons, such that the first lockdown may have been experienced very differently than following lockdowns lasting sometimes over several months. Notions of stress accumulation (Evans et al., 2013) or stress sensitization (Hammen et al., 2000) are in accordance with such a view. Of note then are the implications of the unprecedented collective stressor nature of the pandemic for frameworks that view resilience from a multisystem perspective, i.e., as a process of interaction between various levels of predictors. Namely, that multiple levels of predictors or systems have been affected and depleted simultaneously during the current pandemic, such as social isolation from family systems or depletion of trust in institutions and governments. With multiple systems being, figuratively speaking, “down at once,” it remains unclear what this means for the dynamic process of resilience emerging from the interactions between them.

Relatedly, many models of resilience posit that a range of predictors have an impact on the dynamic resilience-vulnerability process. For example, in trait approaches, components such as mastery or resourcefulness are predictors of resilient responses (Wright & Richmond Mynett, 2019). However, in these approaches, it remains unclear how these predictors function or exert their influence on the resilient outcomes. More recent models have endeavored to provide a distinction between what are considered to be the predictors or factors that facilitate a resilient or a dysfunctional outcome versus what are the mechanisms that drive this facilitation effect (Kalisch et al., 2015). However, in the empirical studies emerging on the impact of COVID-19 on mental health and resilience processes, this theoretical distinction becomes somewhat blurry, as many empirical studies investigate a wide range of mechanisms and predictors in a similar fashion without factoring in the qualitative difference between them. For example, resourcefulness and the coping strategy of seeking support from others are simultaneously posited to be protective factors, but without the distinction that the effect of resourcefulness as a psychological capacity is potentially mediated through the active process of seeking instrumental support. Importantly, during the COVID-19 pandemic many different types of predictors of resilience and vulnerability could have gained relevance for different individuals, depending upon the particular situational and temporal context within the pandemic. Therefore, there is a need for a clearer distinction between influencing predictor factors and socio-emotional or cognitive mechanisms, that is a set of mechanisms through which the impact of the various predictor factors on the resilience-vulnerability process is mediated throughout the pandemic period.

Lastly, two important interrelated aspects, and in our view further drawbacks, which are shared by most conceptual models of resilience-vulnerability and their empirical application is the focus on singular measures at a time of indicators of resilience-vulnerability that are in addition often reduced to a disorder-specific focus on mental health outcomes, such as depression assessed through a single scale. This disorder-specific assessment could range from scores on classic clinical questionnaires such as the Beck Depression Inventory-II (Beck citation) or resilience questionnaires such as the Connor-Davidson Resilience Scale (Connor & Davidson, 2003 citation), or even behavioral, neurobiological, or psychophysiological measures. Such a focus allows for the assessment of only a particular aspect of resilience-vulnerability, which might be unable to provide a more global understanding of the various facets of vulnerability or resilient functioning at any given moment which extend to sub-clinical dimensions of vulnerability or even non-clinical aspects of daily stress or loneliness.

Accordingly, there is growing and converging evidence in support of transdiagnostic approaches to mental health (Dalgleish et al., 2020). Recent frameworks of resilience (Kalisch et al., 2015; Masten et al., 2021a, 2021b) have endeavored to move towards more transdiagnostic and general measurement of resilience and dysfunction in the face of adversity. For instance, the proposition by Kalisch and colleagues (2015) to implement global mental health scores using the General Health Questionnaire (Jackson, 2006) introduced a shift away from the disorder-specific approach to outcome measurement and towards a more transdiagnostic approach in resilience frameworks. However, the increasing prevalence of mental health difficulties such as loneliness, as well as the increasing levels of stress-related burdens and in the pandemic context particularly aspects such as specific mental health burdens, indicate an even broader purview of mental health problems that might not be captured with a particular classical clinical scale or questionnaire, no matter how extensive its scope. Therefore, while theoretical models and empirical studies should certainly be moving forward with a broader idea of psychological difficulties, there is in fact a need to express aggregated resilience and vulnerability on a higher order construct level which integrates multiple indicators of psychological vulnerability ranging from stress, loneliness, situational burdens, anxiety and depressiveness also applicable to non-clinical samples.

## The Wither or Thrive Model of Resilience

Closing these gaps, we propose an integrative novel framework, With:Resilience that is based on the shared strengths of the current resilience models, but at the same time addresses their limitations and extend to the specific features of the current COVID-related pandemic context. The With:Resilience framework is illustrated in Fig. 1, with all its specificities allowing to conceptualize the massive quantities of emerging empirical data about mental health and psychological resilience in times of the COVID-19 pandemic.

In our proposed framework, we conceptualize a plane of functioning that spans resilience and vulnerability as two dimensions of a single higher-level construct, incorporating adaption and maladaptation (Fergusson et al., 2003; Pietrzak & Southwick, 2011), with resilience-vulnerability evolving as a function of time, the presence or absence of stressors, and the influence of time-varying interacting systems of individual, contextual and social factors. The influence of these factors is proposed to be mediated by an array of time- and context-dependent individual regulating mechanisms that are conceptualized in our model as the catalysts that exert the influence of predictors on the resilience-vulnerability outcomes and the process over time. This dynamic resilience-vulnerability is posited to be an outcome of the interaction between various predictors and regulating mechanisms, and the stressor itself or lack thereof, at any given moment. The importance of certain predictors of resilience-vulnerability, and thereby the activation of relevant processing mechanisms, will be dependent upon the current context of the individual at that moment. In case of an existing stressor, the context also includes stressor features. Consequentially, with progress in time and changes in the context of an individual, the pertinent predictors and related processing mechanisms will change, leading to unique expressions of resilience-vulnerability trajectories across time and contexts. This would result in a dynamic process, with an individual oscillating between being more vulnerable at one timepoint, yet tending towards more resilient responses at another. In the following we will discuss each of these single aspects in more detail:

First, as Fig. 1 illustrates, we propose a dynamic model of resilience and vulnerability, that at any given moment, is an outcome of various predictors and mechanisms, and could be represented on a bi-polar dimension: The less vulnerable, the more resilient someone is at a given moment. As stated above, such single higher-level construct enables the integration of multiple highly intercorrelated indicators of psychological vulnerability in the broader population. Accordingly, resilience-vulnerability, which is expressed as a process of functioning in everyday life, cannot be merely measured through single self-report scales or questionnaires that focus on measuring only limited aspects of dysfunction or resilient functioning. Critically, we also propose to incorporate into the construct of resilience-vulnerability aspects that have frequently been found to predict or covary with mental health status such as loneliness, psychological burdens (e.g., conflicts, or feelings of exclusion/discrimination), aggression, and life satisfaction, to name a few. In doing so, we move away from a more specialized clinical focus on resilience-vulnerability, and more towards a generalized or global understanding of resilience-vulnerability that transcends diagnostic categories. This is also important as collective stressors during the pandemic seem to have affected mental health on a broader and larger level, and also becomes apparent in parts of the population which typically are conceived as healthy and not clinically noticeable. However, we do not necessarily imply that current outcome measures are not up to par in explaining partial aspects of dynamic resilience-vulnerability. Instead, our proposal outlines the need for considering resilience-vulnerability to be a higher-order construct that is reflected on various self-report, behavioral, psychophysiological or biological measures, as mentioned above. We suggest that theoretical models and empirical studies should perhaps consider resilience-vulnerability on a more latent level that is expressed as a function of time- and context-varying predictors and processing mechanisms, which likely themselves form constellations on the latent higher-order level.

Furthermore, such an integrated resilience-vulnerability outcome and process can be measured using a variety of tools such as self-report questionnaires, psychological interviews, cognitive-behavioral tasks, biobehavioral measures such as eye tracking or galvanic skin response, physiological measures such as heart rate variability or event-related potentials, biological measures such as epigenetic markers or immune response, and neuroscience methods such as brain imaging.

Second, another important key feature of the With:Resilience model concerns the dynamic and evolving nature of this outcome resilience-vulnerability and what happens to it over time (see *x*-axis of Fig. 1). We propose that the resultant resilience-vulnerability, when combined over multiple timepoints, will be observed as an unfolding process and will manifest in the form of dynamic constellations of aggregated indicators. Our framework especially accounts for the long-term repeated nature of the stressors associated with COVID-19 pandemic which have lasted over several years. Figure 1 proposes four distinct trajectories of resilience-vulnerability as examples: “chronic vulnerability,” “cumulative vulnerability,” “resilient recovery,” and “non-reactive resilience.” These profiles differ both in their dynamic trajectories related to stressor exposure, and in their starting values before the occurrence of a stressor. Figure 1, using different colored lines, shows this important aspect of our model as resilience-vulnerability evolves over the course of time and in response to repeated stressors. The line depicted in blue indicates the non-reactive resilience trajectory, which remains largely stable over time and even in instances of repeated stressor exposure. The line depicted in green indicates the resilient recovery trajectory, which means that dynamic resilience-vulnerability tends more towards dysfunctional responding in the cases of an acute exposure to a stressor. However, individuals following this trajectory would over time show more resilient responding when the stressor become repetitive. In this sense, the resilient recovery trajectory corresponds to the conceptualization of resilience to bounce back in the aftermath of adversities (Block & Block, 1980). The red line depicts the cumulative vulnerability trajectory, which indicates that dynamic resilience-vulnerability will tend more towards vulnerable in case of an acute exposure to a stressor. While individuals in this group will show a return to more resilient responding in the post-stressor recovery period, albeit lesser than the resilient recovery group, however upon repeated exposure they would show an even more pronounced pattern of vulnerable or dysfunctional responding, similar to notions of stress sensitization and stress accumulation (Evans et al., 2013; Hammen et al., 2000). Lastly, the yellow line depicts the chronic vulnerability group, which indicates that dynamic resilience-vulnerability in this group almost always tends towards elevated dysfunctional or vulnerable responding, compared to other groups, despite repetitive exposure to stressors over time. This conceptualization of unique resilience-vulnerability trajectories is in line with the influential work of Bonanno (2004) that proposed four distinct trajectories of “chronicity,” “delay,” “recovery,” and “resilient,” that emerge in the aftermath of a stressor depending upon various individual and environmental predictors. However, importantly, in the With:Resilience framework, these stressor-dependent trajectories are conceptualized not only in the aftermath of an acute or a chronic stressor (Chen & Bonanno, 2020), but also regarding their progress in the face of repetitive exposure to stressors, while accounting for potential recovery periods in between stress exposures.

Third, as can also be seen in Fig. 1, we propose a set of different factors ranging from individual, contextual, demographic, and social factors, along with the processing mechanisms, which influence the outcome resilience-vulnerability. The time-varying and situation-specific nature of many of these factors will varyingly influence the resilience-vulnerability process as it evolves over time. We will discuss each of these different categories in detail below.

Fourth, we differentiate between predicting factors (individual, contextual, demographic, and social factors) and the mediating processing mechanisms through which they exert influence over the dynamic resilience-vulnerability process to enable coping with the stressor. This proposition of our model is in line with the important framework of dynamic resilience processes proposed by Kalisch and colleagues (2015). Thus, in the With:Resilience framework, the influence of specific independent factors must be seen through the lens of how they work in concordance with and through these regulating mechanisms, which are thus mediating the effects of predictors on resilience-vulnerability. We will explain each category of relevant factors in detail in the next subsections.

Fifth, and crucially, not only do the predictors, and the regulating mechanisms through which they function, precipitate the outcome of dynamic resilience-vulnerability at any given moment and its process over time for an individual, they function differentially between persons. In line with other prevailing theoretical models of resilience, the With:Resilience framework posits that different predictors and mechanisms may become salient in different individuals within a given context and at a particular timepoint depending upon the nature of the stressor encountered. As such, this stressor- and context-dependent differential activation of predictors and mechanisms could potentially lead to varying outcomes of dynamic resilience-vulnerability for different individuals. Therefore, distinct potential trajectories of dynamic resilience-vulnerability process will likely emerge as a result.

In sum, the proposed model accounts for (1) multi-method approaches to resilience and vulnerability, (2) the dynamic nature of resilience-vulnerability processes over time, (3) a variety of factors that influence resilience-vulnerability through (4) different processing mechanisms, and (5) interindividual as well as situational and context-specific differences. In the following we discuss in more detail all the different categories of influencing factors we integrate in our With:Resilience Model.

### Individual Psychological and Biological Factors

A key set of predictors that influence the resilience-vulnerability outcome, and eventually the process over time, are factors that are unique to an individual, which can be characterized as relatively stable characteristics, and some of which can even be innate and inherent in nature. This category of predictors encompasses all biological, psychological and historical aspects that are specific to an individual and that impact the particular way an individual may respond to stressors. For example, a person high on neuroticism or with a history of childhood maltreatment is considered at risk of an adverse resilience-vulnerability trajectory (Sonuga-Barke et al., 2017). As such, this category of predictors can be further divided into biological factors, psychological factors, and individual life experience factors. A variety of biological factors has been proposed to influence the process of psychological resilience and vulnerability. Some of these factors include inflammation markers (Wang et al., 2018), various genetic markers such as FKBP5 gene (Feder et al., 2009; Russo et al., 2012), resting state connectivity (Workman et al., 2017), brain-derived neurotrophic factor (Brown et al., 2020; Rothman & Mattson, 2013), autonomic nervous system (Pereira, Campos & Sousa, 2017), hypothalamic pituitary adrenal (HPA) axis (Baumeister et al., 2014), glutamate (Reus et al., 2018), endocannabinoids (Maldonado et al., 2020), and structural brain abnormalities (Dedovic et al., 2009). Additionally, the physical health status of an individual, such as suffering from a chronic or acute disease, and lifestyle factors, such as diet and level of physical exercise, can serve as important biologically related predictors of the resilience-vulnerability (Alexandratos et al., 2012; Bremner et al., 2020; Carreira et al., 2018; Goubert & Trompetter, 2017; Pham et al., 2019).While neurobiological factors specific to an individual have been shown to affect the level of resilience-vulnerability of a person, similarly a range of psychological trait-like characteristics have also been posited to play a key role. To name a few, psychological factors like neuroticism (Oshio et al., 2018), attachment style (Atwool, 2006), optimism (Boldor et al., 2012), self-esteem (Johnson et al., 2017), self-efficacy and locus of control (Dunn et al., 2007), mindfulness (Thompson et al., 2011), self-compassion (Mona & Angela, 2018; Neff & McGehee, 2010), temperament (Condly, 2006), intolerance of uncertainty (Einstein, 2014), and cognitive biases such as interpretation or attention bias (Derakhshan, 2020) can influence resilience-vulnerability. Lastly, in addition to biological and psychological factors, the unique life experiences of an individual also shape the way how individual will respond in the face of present and future stressors. This happens by the way of parenting (Black & Lobo, 2008) or exposure to early life adversity (Méndez Leal & Silvers, 2021). Along the lines of cumulative-events approaches to stress resilience (Seery et al., 2010), even traumatic experiences which themselves have been stressors at early points in time in the life of an individual, and their personal history of mental health difficulties could serve as potential predictors of differential time courses in resilience-vulnerability outcomes.

### Contextual and Demographic Individual Factors

In addition to individual biological and psychological factors, there is surmounting evidence that the context and the demographic status of an individual also influence resilience-vulnerability. Age and sex are important demographic predictors, with a plethora of studies showing that being young or having female sex are risk factors for developing mental health difficulties (Masten, 2002; Tolin & Foa, 2008). Furthermore, socioeconomic factors such as household income and employment status, i.e., whether an individual is employed full time or marginally, influence physical and mental health outcomes and even the developmental trajectories of an individual Hergenrather et al., 2015; Kawakami et al., 2012; Reiss, 2013; Sareen et al., 2011). Relatedly, the conditions of employment, such as working hours, conditions at workplace and supervision, are found to be associated with prediction of mental health outcomes and therefore serve as an important contextual factor for resilience-vulnerability (Modini et al., 2016; Rönnblad et al., 2019). Moreover, an individual’s educational status influences resilience-vulnerability, with individuals having low levels of education being more prone to poorer mental health outcomes while high levels of education serve as a protective factor (Silva et al., 2016). Furthermore, an individual’s household conditions can influence resilience-vulnerability, with living alone and being a single parent serving as predictors of mental health problems (Umberson et al., 2013). Some of the lesser studied contextual predictors of resilience-vulnerability can be living conditions such as housing conditions or sufficient space in dwelling (Evans et al., 2003). An increasingly important contextual variable associated with resilience-vulnerability, especially in urban areas, seems to be the access to green spaces, although concerted research efforts in this area are still limited and therefore not entirely conclusive (Gascon et al., 2015; A. C. K. Lee & Maheswaran, 2011). Another key demographic predictor of resilience-vulnerability is the sexual orientation of an individual, with sexual minorities often reporting some of the poorest mental health outcomes (Plöderl & Tremblay, 2015). Furthermore, despite there being a dearth of empirical studies exploring the mental health of immigrants, migratory background can serve as a potential predictor of resilience-vulnerability. Immigrants report noticeable mental health difficulties (Bas-Sarmiento et al., 2017), and these become especially pronounced within the context of refugees (Bogic et al., 2015). Moreover, a key contextual factor predicting resilience-vulnerability processes would be cultural factors, such as identification with an ethnic group, being part of cultural majority in a location, or experiences of social justice, to name a few (Anderson, 2019; Dennis et al., 2010; Han et al., 2016; Raghavan & Sandanapitchai, 2020). While some frameworks have proposed the significance of cultural factors in explaining resilience-vulnerability processes (Ungar, 2013), there has been little work addressing this crucial set of predictors, and this gap would need to be addressed to have a complete understanding of resilience-vulnerability process interculturally. Lastly, a critical contextual factor with respect to the pandemic will be the covid-specific factors that influence resilience-vulnerability processes. This would include variables that are specific to the pandemic and would impact resilience and vulnerability processes, such as being at a biologically increased risk for contracting COVID-19 such as chronic illnesses (Louvardi et al., 2020), having access to vaccination against COVID-19 disease (Perez-Arce et al., 2021), or being in a type of employment that poses an increased risk of contracting the COVID-19 disease such as being a system-relevant worker (Cai et al., 2020; Xu et al., 2020). Moreover, pandemic-specific worries such as resource scarcity or pandemic-specific burdens such as increased childcare burden that have been shown to influence mental distress will also comprise the covid-specific factors category and will be crucial to determining the resilience-vulnerability trajectory (Zheng et al., 2021). Importantly, we must keep in mind that most of these contextual and demographic factors are often found to be interacting with, and precipitating and reinforcing, each other.

### Social Intersubjective Factors

In addition to psychological, biological, demographic, and contextual individual factors, factors that govern how an individual socially interacts with others, whether they are single individuals, groups or even institutional social contacts, could also predict how a person will differentially respond to adversity. An important social predictor of resilience-vulnerability, which has been the subject of wide empirical investigative efforts, is the level of social support received and perceived by an individual, whether it be functional, instrumental, emotional, etc. (Southwick et al., 2016). However, on the level of individuals, perceived social support, and its various iterations, is only one of the factors forming a larger set of social resources, including an individual’s sense of belonging to their social surroundings, or social networks and interactions (Silveira et al., 2022), as well as social capacities such as levels of empathy, compassion and mentalizing ability or our level of prosocial engagement and motivation (Singer et al., 2004). Despite the recognition of resilience enabling properties of selected social factors in prevailing resilience frameworks (Masten et al., 2021a, 2021b; Michael Ungar & Theron, 2020), particularly social competencies and capacities have not found a consistent and conspicuous place in prevailing models of psychological resilience and vulnerability yet, and thereby empirical investigation at large. However, we propose that these aspects are also key to predicting whether an individual tends more towards a vulnerable or resilient function (Kim, 2020). Initial empirical studies are providing evidence that such social competencies can serve as protective factors for mental health and could be crucial targets for interventions aimed at enhancing resilience (Borkowska & Laurence, 2021; Corvo & de Caro, 2020). Social competencies such as empathy and compassion, and their training, have been found to have a protective effect on mental health (Kinman & Grant, 2011; Spandler & Stickley, 2011).

Other than social capacities, we uniquely propose that dynamic resilience-vulnerability process over time could be impacted by a group of factors and processes that fall under the umbrella of the concept of *social cohesion* frequently used in the social and economic sciences. Social cohesion is the extent to which various individuals and groups within the society are connected with each other (Manca, 2014). Social cohesion factors signal the degree to which a society can be characterized by togetherness and its hallmark features concern social interaction and inclusion, civic engagement and identity, social structures, norms and value systems that promote loyalty and solidarity, human rights, interindividual and institutional trust, conflict management, the absence of crime and lack of social or economic inequalities (Chan et al., 2006; Schiefer & van der Noll, 2017). In line with the multisystems perspective of resilience, social cohesion factors are proposed to comprise of multiple systems, micro (e.g., families, relationships), meso (e.g., communities, neighborhoods, institutions), and macro (e.g., nations) systems of a society (Fonseca et al., 2019; Friedkin, 2004). Importantly, social cohesion has also been posited to be multidimensional in nature, i.e., it concerns cross-sectional horizontal cohesion between individuals, communities, and institutions, but also reflects on a vertical dimension between the citizens and a state (Chan et al., 2006; Fonseca et al., 2019). Social cohesion has been proposed to influence individual functioning through the adoption and reinforcement of health-related behaviors and through increased access to resources (Kawachi & Berkman, 2000). Furthermore, social cohesion has been proposed to aid mental health outcomes in disasters by providing capacities for psychological processing through meaningful contact with others and enhanced feeling of sense of purpose and connection (Greene et al., 2015; Silver et al., 2002). In instances of adverse situations that affect the community as a whole, such as the present pandemic context, greater levels of social cohesion should likely generate greater interaction and communication, which would enhance feelings of togetherness and augment problem-solving abilities (Greene et al., 2015). However, given the unprecedented social isolationary nature of the current pandemic, it would be these exact social cohesion parameters that will likely be adversely affected leading to perhaps more vulnerability and less resilient functioning. Therefore, we propose to incorporate in the With:Resilience model, a set of social cohesion factors that could be decisive for the dynamic resilience-vulnerability outcome and process.

One social cohesion factor that has seen wide empirical efforts in the context of mental health and resilience revolves around social network properties. This line of research has indicated that social network size, intensity, and changes in the network could be crucial factors for the resilience-vulnerability process over time (Levula et al., 2016; Youm et al., 2014). Another prominent social cohesion factor is belongingness. Belongingness or a sense of belonging to one’s surroundings and to social groups such as peers and friends, and to geographical units, such as one’s city or country, could serve as a protective factor for mental health (Baskin et al., 2010; Miao et al., 2021; Scarf et al., 2016; Shakespeare-Finch & Daley, 2017). Furthermore, frequency of prosocial behavior towards other and experience of prosocial behavior from others has also been shown to be a protective predictor for mental health processes (Haroz et al., 2013; Raposa et al., 2016). Moreover, feelings of trust also form a crucial component of social cohesion as it forms the basis of social capital, along with social engagement and participation which is also a foundational aspect of social cohesion (Kosfeld et al., 2005; Putnam, 1993). Trust is seen as an individual’s feeling or expectation of probability or predictability of behavior of others behavior as well as the intentions of the individual (Glaeser et al., 2000). Given the association of trust to social affiliation and attachment, it could potentially influence the resilience-vulnerability outcomes of an individual during stressful times. A recent study (Silveira et al., 2022) found trust to be negatively associated with vulnerability while being associated with higher trait resilience. A key concept in social cohesion, interpersonal synchrony, which can be defined as the overlapping movements of two or more individuals in time, has been found to be associated with positive affect, reduced stress and positive behavioral outcomes such as increased helping behavior, and thus could prove to be a compelling contributor to resilience-vulnerability processes over time (Göritz & Rennung, 2019; Rennung & Göritz, 2016). Finally, perceived and instrumental economic inclusion, which is a crucial concept in the social cohesion literature but is more discussed within the framework of resilience to disasters, could also serve as a potentially crucial predictor of resilience-vulnerability (Every & Thompson, 2014). As becomes evident, more empirical work is needed to clarify the nature and scale of the impact of these social cohesion predictors on resilience and vulnerability.

## Processing Mechanisms

While individual psychological, biological, contextual, and demographic factors as well as social intersubjective factors influence the way how someone will respond to stressful events and life-adversity, these factors do not necessarily function in isolation but have to exert their influence through a range of cognitive or affective information processing mechanisms or biological regulating mechanisms. As such, specific information is processed through specific computations or mechanisms by the individuals and these modulate and shape the effects of the above-mentioned factors on the resilience-vulnerability outcomes. These, largely adaptive or regulatory capacities have been conceptualized as mechanisms that mediate developmental impacts of, the much more passive in nature, predictive factors on the resilience-vulnerability outcomes and time courses (Kalisch et al., 2017). In other words, the factors explain why an individual develops mental dysfunction when faced with stressors while others do not, but the mechanisms explain how the dysfunction or healthy function comes to be. These processing and regulating mechanisms, when engaged in the face of a stressor, allow an individual to return to a state of homeostasis and psychological balance that had been disturbed by the presence of the stressors, perhaps leading to a temporary psychological dysfunction (Masten & Motti-Stefanidi, 2020). In other words, this set of individual processing and regulating mechanisms in fact can harness the strengths of the various predictors to produce a resilient response in the face of and in the aftermath of a stressor. These processing mechanisms can be observed on different levels, ranging from biological, neural, to psychological socio-emotional and socio-cognitive processes and computations. Some of these biological regulation mechanisms include epigenetic changes such as methylation (Dudley et al., 2011), cellular changes such as neurogenesis (McEwen & Gianaros, 2011), and subcellular changes (Leyton, 2007). Several studies have also indicated the adaptive nature of neural mechanisms such as synaptic modifications and brain plasticity in precipitating resilience (Maier, 2015; Wang et al., 2014). Moreover, studies have also indicated that physiological mechanisms such as regulation of one’s autonomic system, expressed through changes in vagal tone or cardiac autonomic regulation, also serve as a regulating mechanism in instances of stressors (Dedoncker et al., 2021; Thayer et al., 2012).

Apart from these biological and neural mechanisms, some of the most widely researched cognitive and behavioral processing mechanisms within the context of stress resilience and vulnerability concern the use of coping and emotion regulation strategies such as using humor as a way to cope, positive cognitive and emotional reappraisal of situations, acceptance of emotions and difficult situations, distraction and denial (Abel, 2002; Booth & Neill, 2017; Gross & John, 2003; Kohl et al., 2012; McCain et al., 2018; Meneghel et al., 2019; Rzeszutek et al., 2017; Thompson et al., 2018; Troy & Mauss, 2011). Some studies operationalize these strategies to be predictors of resilience-vulnerability or even as direct indicators of resilience itself. For example, emotion regulation strategies such as rumination or acceptance are often investigated interchangeably as predictors, mechanisms and outcomes of resilience-vulnerability. In the With:Resilience model, we suggest that such processing mechanisms can modulate the impact of a certain predictor on the resilience-vulnerability outcomes and time courses. For example, someone high on intolerance of uncertainty when employing a regulation mechanism of rumination in a stressful situation could end up displaying a vulnerable response; however, the same individual when employing a positive reappraisal regulation mechanism in a stressful situation will perhaps display more resilient functioning. As such, operationalizations of those strategies as predictor or indicator of resilience are unable to explain why particular strategies are evident in some stress situations, while others in another type of stress situation. Furthermore, some studies also demarcate adaptive from maladaptive strategies (Brown et al., 2005; Holton et al., 2016). However, these views have been challenged by recent models of self-regulation which propose that the adaptive nature of regulating mechanisms is dependent upon the stressor context, including the nature and intensity of the stressor (Aldao, 2013). For example, rumination, which is proposed to be largely maladaptive could in fact prove to be helpful when focusing on negative feedback from one’s boss to improve work performance, in combination with other strategies such as problem-solving, potentially leading to long-term positive consequences for the individual. Therefore, depending upon the context and time, the application and execution of coping and regulation strategies changes. Importantly, this context-dependent implementation of coping and emotion regulation strategies needs to function in conjunction with cognitive mechanisms such as flexibility and cognitive control which involve switching and inhibition of various cognitive processes (Aldao et al., 2015; Pruessner et al., 2020; Sanchez-Lopez et al., 2019). This indicates that individuals, depending upon the stressor context, will need to flexibly switch between their repertoire of coping and regulation strategies, and activate some while inhibit other strategies in a particular situation to yield the maximum adaptive effect within a context (Sanchez-Lopez, 2021), leading to the development of the resilience trajectory.

Within the context of the current COVID-19 pandemic, many studies have examined the use of coping strategies to counteract stressful situations (Bendau et al., 2021; Panayiotou et al., 2021; Prati, 2021), but only a minute number have looked at flexible use of coping and regulation strategies (Shabat et al., 2021) finding differential use of strategies depending upon the individual and their context. Although the flexible application of our proposed processing mechanisms, including coping and regulation strategies along with the biological and neural mechanisms, within the pandemic context needs further investigation, it becomes evident that these processing mechanisms modulate the influence of the various predictor factors to lead to resultant resilient or vulnerable functioning. The mediation of predictors by shifting processing mechanisms over time then leads to the emergence of a dynamic process of resilience-vulnerability, as the outcome resilient or vulnerable function also changes over the time course. Crucially, given the individual differences that exist in presence of range of predictors and implementation of processing mechanisms, we then expect divergence in the resilience-vulnerability process in terms of the manifestation of unique trajectories of resilience-vulnerability (Ahrens et al., 2021).

## Perspectives on the With:Resilience Model

Now that we have expanded on the various elements of the Wither or Thrive model of Resilience, in this section, we will discuss the immediate and long-term applications and implications emerging from our model including methodological implications.

### Methodological and Statistical Recommendations

As is evident from the discussion of the framework in the above sections, the With:Resilience model allows direct and immediate application to the pandemic context in terms of aggregating empirical data that has been collected in the context of the COVID-19 pandemic.

Firstly, the current pandemic is reflective of an accumulation of risk and vulnerability over time, with repeated lockdowns and consecutive waves of COVID-19 infection. Moreover, as mentioned previously, with multiple systems affected at once there is an opportunity for manifold increase which will likely aggregate over time (Masten & Motti-Stefanidi, 2020). With repeated occurrences of these stressors, capacities to recover and adapt may become severely depleted over time leading to a cumulative pandemic fatigue effect (Haktanir et al., 2021; Singer et al., 2021). However, such progressive changes can only be assessed by the use of longitudinal designs with repeated measurements of resilience-vulnerability markers and methodologies that then can exploit the resulting different time courses emerging over time (Prime et al., 2020), especially considering that predictors and processing mechanisms themselves are proposed to be time-varying as well. Such longitudinal time-courses can be tested directly using growth curve models to identify evolution of dynamic resilience-vulnerability over time (McArdle & Nesselroade, 2003). Moreover, as we propose, evolution of dynamic resilience-vulnerability process would likely take a different trajectory for different groups of people, latent class analyses can be used to understand which particular class or trajectory an individual time-course of dynamic resilience-vulnerability takes over time (Nylund-Gibson & Choi, 2018).

Secondly, the With:Resilience model proposes to move away from single-scale approaches and towards conceptualizing resilience and vulnerability as a broad scoped higher order concept that encompasses various aspects of vulnerability and resilience that would not usually be considered, but are important in the pandemic context. Therefore, analytically, we recommend the use of structure equation modeling approaches to derive aggregated latent factors which are reflective of the various measures of resilience-vulnerability over time, with a latent factor emerging at every timepoint of longitudinal assessment (Lee et al., 2011 for example). This would also allow to clarify if different latent factor structures for the latent construct of resilience-vulnerability emerge over different time points during the course of the pandemic.

Thirdly, our framework proposes a range of risk and protective factors, and the potential mechanisms through which they engender on dynamic resilience-vulnerability time courses. Such proposals can be tested directly using growth mixture models and latent class analysis to understand which predictors and processing mechanisms might be influencing different classes of trajectories proposed by the model. However, given the very wide range of predictors and processing mechanisms proposed within the framework, another highly suitable approach for testing the emergence of trajectories of resilience-vulnerability owing from predictors and mechanisms might be the use of machine learning methods such as recursive partitioning (Berman & Hegel, 2014). Moreover, application of the use of cross-lagged models might be helpful in delineating the predictor-mechanism combinations and the resultant resilience-vulnerability trajectories, isolating directional influences.

Existing cohort studies, in which data on certain parameters of resilience-vulnerability had already been collected prior to the pandemic, and which now extend to longitudinal assessments during the pandemic might prove to be especially enlightening. Crucially, given the time-varying nature of many of the proposed predictor constellations, regulating mechanistic capacities, and the resilience-vulnerability process over time, taking a dynamic network approach or complex systems approach to analyses might also be useful, particularly to map predictor-mechanism constellations in their impact on changes in networks of dynamic resilience-vulnerability constructs (see Kalisch et al., 2019 for a dynamic network perspective of resilience and psychiatric disorders).

### Policy Implications

There are also several policy implications associated with our model. First and foremost, our framework implores our policymakers to not undertake imposition of social isolation measures, such as lockdowns, without considering the requirements of various populations and sub-groups, such as the cumulative vulnerability trajectory group that is proposed in our model. Such individuals, with time, keep tending towards increased vulnerability, and require specific attention in the lead up to lockdowns. As such, policymakers can make use of our model to understand which groups of individuals, depending upon which combinations of predictors, would be worse off in instances of repeated stressors such as lockdowns and need most protection and active support. Although our framework might seem as largely precautionary in nature, it in fact also provides ways in which policymakers can help build back a stronger and a more resilient society (Aknin et al., 2021). This can be accomplished through, through identification of various person-specific psychological, contextual, or demographic aspects and social life of the general population to identify the most vulnerable sub-groups which need most protection. It can further be accomplished through empowering local support groups, mental health organizations and volunteer groups. Similarly, local networks can be empowered to provide the right set of interventions to the people who need them. This would also ease the burden on clinicians and practitioners.

### Practical Interventional Implications

In addition to the above discussed methodological and policy implications emerging from our framework, there are some important practical ramifications associated (Joyce et al., 2018). Our framework brings to fore the importance of factoring in the entire holistic ensemble of factors, ranging from an individual’s biopsychosocial makeup to the inter-individual factors and individual context, to understand who could benefit from interventional support during a pandemic. Within this context, our framework can provide a theoretical basis and guide for clinicians and practitioners to understand which of their patients could benefit from an intervention. Our framework might also theoretically assist interventional work aimed at informing who might benefit from what type of intervention at what timepoint, and whether repeated applications of intervention and what intensity of intervention might be needed.

Our framework also indicates a need for developing novel interventions to target unique aspects that might influence the resulting dynamic resilience-vulnerability. Some factors are easier to intervene with, for example, lifestyle factors such as diet and exercise can be easily moderated to influence resilience-vulnerability. However, other factors such as social intersubjective factors are trickier to intervene since they need a whole host of empirical work to design interventions that modulate these factors.

The fact that our framework also highlights processing mechanisms that may be adaptive and regulative in mediating the influence of these factors on resilience-vulnerability outcomes will likely also help develop targeted intervention fostering these specific socio-emotional or cognitive processes through mental training (Berking et al., 2008; Cameron et al., 2007; Hildebrandt et al., 2019; Roemer et al., 2015). Accordingly, our framework also highlights the need to move towards the final frontier of precision interventions that can be developed using our model as the guiding framework (Bidargaddi et al., 2020; Purgato et al., 2021). Precision interventions for mental health would aim to provide interventional support for particular predictors and mechanisms at a certain time point, which then evolves to supporting other predictors and mechanisms at other timepoints, keeping in view also the time-dose effects. Such precision interventions can receive an empirical thrust through our framework which takes a comprehensive, time- and context-sensitive approach needed for the development of such interventions. Interestingly, the current pandemic has put a special focus on the development of scalable online interventions that can be applied to the general population at large (Figueroa & Aguilera, 2020). Future empirical work aimed at developing novel and refining existing interventions for online application can also leverage the With:Reilience framework to define their objectives in terms of predictors and mechanisms being targeted, and can also benefit from the broad approach to dynamic time courses of resilience-vulnerability taken in this model.

## Conclusion

The Wither or Thrive model of Resilience, With:Resilience, aims to provide an overarching framework which integrates many aspects of previous psychological resilience frameworks into one unifying model and at the same time helps close important gaps that become apparent within the current pandemic context and enables a more comprehensive understanding and analytical modelling of all the incoming data on mental health and resilience during the COVID-19 pandemic.

Firstly, and crucially, the With:Resilience framework introduces a broader conceptualization of resilience and vulnerability on a dynamic plane of functioning, based on not just singular clinical scales but represented on a higher-order level. Specifically, we conceptualize resilience-vulnerability as a higher-level latent construct comprising of multiple indicators with resilience being on the one pole and vulnerability on the other pole of one dimension. Importantly, we also include non-traditional indicators of vulnerability such as loneliness, stress and different types of psychological burdens. As such, we echo a more inclusive view that accounts for the ways in which vulnerability manifests in the general populace in the current pandemic, not restricting it only to clinical outcomes.

Secondly, it outlines several different trajectories of resilience-vulnerability over time that emerge during instances of different and repeated stressors over longer duration of time (over years). By doing this, it can, for example, account for different effects such as first lockdown shock effects (potentially observed after the first shorter lockdowns) or lockdown fatigue effects (observed in further lockdowns extending over many months) and identify how different types of people can respond to such repeated collective stressors over time in different ways: some more resilient than others.

Thirdly, the framework proposes a rather comprehensive set of factors ranging from psychological, biological, contextual and demographic individual factors as well as social intersubjective factors including social capacities and social cohesion markers. All these factors act as predictors influencing the different trajectories of resilience-vulnerability over time.

Fourthly, the model proposes another class of variables as mediators between prediction factors and the classes of different trajectories of resilience-vulnerability outcomes. These processing mechanisms represent neurobiological, psychophysiological, socio-emotional as well as socio-cognitive regulating mechanisms such as epigenetic changes, autonomic regulation mechanisms, emotional regulations strategies, cognitive flexibility, appraisal, attributional and perceptual styles as well as social skills that in turn allow for social support strategies. As these intermediate processing mechanisms mediate whether specific factors will have detrimental effects on mental health or not, it is there where intervention research can most efficiently elicit adaptive changes.

We further propose statistical methods of how to adequately model the complex interaction between influencing factors, processing mechanisms onto different classes of time trajectories of resilience-vulnerability outcomes. Importantly, the framework also has implications for public health policies and policymakers with respect to imposition of repeated long-term social isolation measures.

In sum, the With:Resilience model provides an important theory-driven, integrative framework with a dynamic view on resilience and vulnerability that allows for appropriate modeling of complex longitudinal pandemic-related data, and helps identify which factors allow people to wither or to thrive when exposed to unprecedented, repeated and continuous stressors. As countries around the globe continue to battle with the fourth and the fifth waves of the pandemic with further lockdowns and social isolation orders, such a model will help to integrate existing datasets, help choose the adequate methodologies to model these complex data evolving over time, and assist in the development of actionable guidelines for society as well as the clinical sciences that are necessary to protect the most vulnerable amongst us.

## Data Availability

The manuscript has no associated data.
